# Evaluation of the results of patients who underwent coronary bypass grafting with or without cardiopulmonary bypass pump

**DOI:** 10.1186/1749-8090-8-S1-O188

**Published:** 2013-09-11

**Authors:** P Kwinecki, T Stankowski, A Kędziora, A Pawęzowska, SS Aboul-Hassan, R Cichoń

**Affiliations:** 1 Heart Diseases Center MEDINET, Wroclaw, Poland

## Background

The type of treatment can be chosen depending on the amount, localization and the advancement of stenosis in the coronary arteries. Aim of this study is to compare the results of surgical treatment of ischemic heart disease using the extracorporal circulation (ECC) - CABG or without the ECC - OPCAB.

## Methods

In this study 616 patients were evaluated (78% males, 22% females, Euroscore: 4.8±2.73, age: 65±8.91 years old). 1st group - 316 patients (75% males, 25% females, Euroscore:4.9±2.77) underwent OPCAB was compared with 2nd - group who underwent CABG (300 patients, 81% males, 19% females, Euroscore: 4.7±2.69) in terms of the amount revascularized vessels, amount of reoperations caused by postoperative bleeding, amount of postoperative blood loss, amount of blood products transfused and postoperative complications.

## Results

Duration of OPCAB- 2hrs28min± 34.63min, CABG- 2hrs54min±34.28min (p<0.001). Average amount of vessels which underwent revascularization in the OPCAB - group was 2.3, in the control group was 2.9 (p=0.003). Average amount of postoperative drainage 18 hours after the surgery:1st group 566ml vs 760 ml (p<0.001). Average amount of transfused blood products:PRBC - 1st group 0.7U vs 1.7U (p<0.001); Platelets - 1st group 0.5U vs 1.2U (p<0.001); Plasma - 1st group 0.6U vs 1.5U (p<0.001). Rethoractomy caused by postoperative bleeding: 1st group 1.26% vs 3.67% (p=0.05). Major Adverse Cardiac and Cerebrovascular event (MACCE) in: 1stgroup 3.1% vs 3.0% (p>0.05).

## Conclusion

Despite the statistically significant difference of number of grafts per patient, OPCAB procedure providesreduced duration of the surgery, postoperative drainage and transfused blood products. The similar MACCE amount in both groups suggests that the OPCAB revascularization was efficient.

